# Genomic Insights into Niche Partitioning across Sediment Depth among Anaerobic Methane-Oxidizing Archaea in Global Methane Seeps

**DOI:** 10.1128/msystems.01179-22

**Published:** 2023-03-16

**Authors:** Jiawei Chen, Yingdong Li, Cheng Zhong, Zhimeng Xu, Guangyuan Lu, Hongmei Jing, Hongbin Liu

**Affiliations:** a Southern Marine Science and Engineering Guangdong Laboratory (Guangzhou), Guangzhou, China; b Department of Ocean Science, Hong Kong University of Science and Technology, Hong Kong, China; c Department of Ocean Science and Hong Kong Branch of the Southern Marine Science and Engineering Guangdong Laboratory (Guangzhou), The Hong Kong University of Science and Technology, Hong Kong, China; d CAS Key Laboratory for Experimental Study under Deep-sea Extreme Conditions, Institute of Deep-sea Science and Engineering, Chinese Academy of Sciences, Sanya, China; e Research Center for the Oceans and Human Health, City University of Hong Kong Shenzhen Research Institute, Shenzhen, China; University of Hawaii at Manoa

**Keywords:** ANME archaea, AOM, niche partitioning, methane seep, metagenome-assembled genome

## Abstract

Marine sediments are important methane reservoirs. Methane efflux from the seabed is significantly restricted by anaerobic methanotrophic (ANME) archaea through a process known as anaerobic oxidation of methane (AOM). Different clades of ANME archaea occupy distinct niches in methane seeps, but their underlying molecular mechanisms still need to be fully understood. To provide genetic explanations for the niche partitioning of ANME archaea, we applied comparative genomic analysis to ANME archaeal genomes retrieved from global methane seeps. Our results showed that ANME-2 archaea are more prevalent than ANME-1 archaea in shallow sediments because they carry genes that encode a significantly higher number of outer membrane multiheme *c*-type cytochromes and flagellar proteins. These features make ANME-2 archaea perform direct interspecies electron transfer better and benefit more from electron acceptors in AOM. Besides, ANME-2 archaea carry genes that encode extra peroxidase compared to ANME-1 archaea, which may lead to ANME-2 archaea better tolerating oxygen toxicity. In contrast, ANME-1 archaea are more competitive in deep layers than ANME-2 archaea because they carry extra genes (*mtb* and *mtt*) for methylotrophic methanogenesis and a significantly higher number of *frh* and *mvh* genes for hydrogenotrophic methanogenesis. Additionally, ANME-1 archaea carry exclusive genes (*sqr*, *TST*, and *mddA*) involved in sulfide detoxification compared to ANME-2 archaea, leading to stronger sulfide tolerance. Overall, this study reveals the genomic mechanisms shaping the niche partitioning among ANME archaea in global methane seeps.

**IMPORTANCE** Anaerobic methanotrophic (ANME) archaea are important methanotrophs in marine sediment, controlling the flux of biologically generated methane, which plays an essential role in the marine carbon cycle and climate change. So far, no strain of this lineage has been isolated in pure culture, which makes metagenomics one of the fundamental approaches to reveal their metabolic potential. Although the niche partitioning of ANME archaea was frequently reported in different studies, whether this pattern was consistent in global methane seeps had yet to be verified, and little was known about the genetic mechanisms underlying it. Here, we reviewed and analyzed the community structure of ANME archaea in global methane seeps and indicated that the niche partitioning of ANME archaea was statistically supported. Our comparative genomic analysis indicated that the capabilities of interspecies electron transfer, methanogenesis, and the resistance of oxygen and hydrogen sulfide could be critical in defining the distribution of ANME archaea in methane seep sediment.

## INTRODUCTION

Methane is an essential greenhouse gas, contributing ~20% to global warming since the postindustrial period (1). Most methane on earth is biogenic through methanogenesis, which occurs in various anoxic subsurface environments (2). Since the ocean covers ~70% of the earth’s surface, marine sediments are one of the largest methane reservoirs, which deposit 450 to 2,000 gigatonnes (Gt) of methane-bound carbon (Gt C) and produce 0.085 to 0.3 Gt C annually (3, 4). Approximately 0.02 Gt C stored in the subsurface seabed of continental margins seeps into the seafloor annually due to gravitational and tectonic forces (5). However, only <2% of the global methane flux is contributed by the ocean. This is because ~90% of the methane produced in deep marine sediments is consumed before it reaches the seafloor by anaerobic methanotrophic (ANME) archaea through a process known as anaerobic oxidation of methane (AOM) (3).

AOM is mediated by microbial consortia of ANME archaea and bacteria. The former oxidize methane to CO_2_ via a reverse-methanogenesis pathway, whereas the latter reduce sulfate, metal, and nitrate/nitrite (1, 6). AOM coupled to sulfate reduction (AOM-SR) is the main biological sink of methane in marine sediments because sulfate is the dominant anion at the marine sediment-water interface (6). Therefore, the main niche for ANME archaea and the associated sulfate reduction bacteria (SRB) is the sulfate-methane transition zone (SMTZ) in marine sediments, where the upward-diffusing methane meets the downward-transported sulfate from seawater. Remarkably, in deep SMTZ and layers below it, AOM often intertwined with methane production (MP), and all potential methanogens in these layers also belonged to ANME archaea (7–9).

ANME archaea can be grouped into three distinct clades, including ANME-1, ANME-2, and ANME-3, according to their phylogenetic relationships based on 16S rRNA genes (1) as well as genome-wide analysis (10). Fluorescence *in situ* hybridization (FISH) analysis shows that ANME-1 archaea are often observed as single rod-shaped cells or in chains of a few cells, while most ANME-2 and ANME-3 archaea form coccoid consortia with SRB (1, 6). Different clades of ANME archaea occupy distinct niches and geographic distributions (1). ANME-1 and ANME-2 are the dominant ANME archaeal clades and often co-occur in most methane seep sediments, whereas ANME-3 archaea appear to be restricted to some mud volcano ecosystems and are only occasionally observed. Remarkably, a niche partitioning was observed between ANME-1 and ANME-2 archaea related to the depth below the seafloor in methane seep sediments. ANME-2 archaea often predominate the AOM communities in a shallow SMTZ, while ANME-1 archaea prefer to populate deep sediment layers (11–17). However, the molecular mechanisms underlying their niche partitioning are not fully understood.

Considering the benthic AOM exhibits efficient restriction of methane efflux and its significance in the carbon cycle and global warming, investigating the environmental factors controlling the distribution of ANME archaea in marine sediments can help us to understand the ocean’s role in climate change. So far, no ANME archaeal strain has been isolated in pure culture, making metagenomics one of the fundamental approaches to studying their metabolic potential. Since ANME archaea are naturally enriched in methane seep sediments (1, 6), which facilitates the recovery of high-quality metagenome-assembled genomes (MAGs), and environmental factors vary significantly in methane seep sediments on spatial scales of several centimeters below the seafloor (1, 5), methane seep can be an ideal ecosystem to study the genomic flexibility of ANME archaea and their controlling stressors. In this study, we reviewed the distributional pattern of ANME archaeal communities in global methane seeps and verified their vertical niche partitioning in sediments. Furthermore, we compared ANME archaeal MAGs recovered from global methane seeps and identified genomic flexibility among them. This work aims to reveal that genomic adaptation is a fundamental force in shaping the niche partitioning of ANME archaea.

## RESULTS AND DISCUSSION

### Niche partitioning among ANME archaea in global methane-seep sediments.

**FIG 1:**
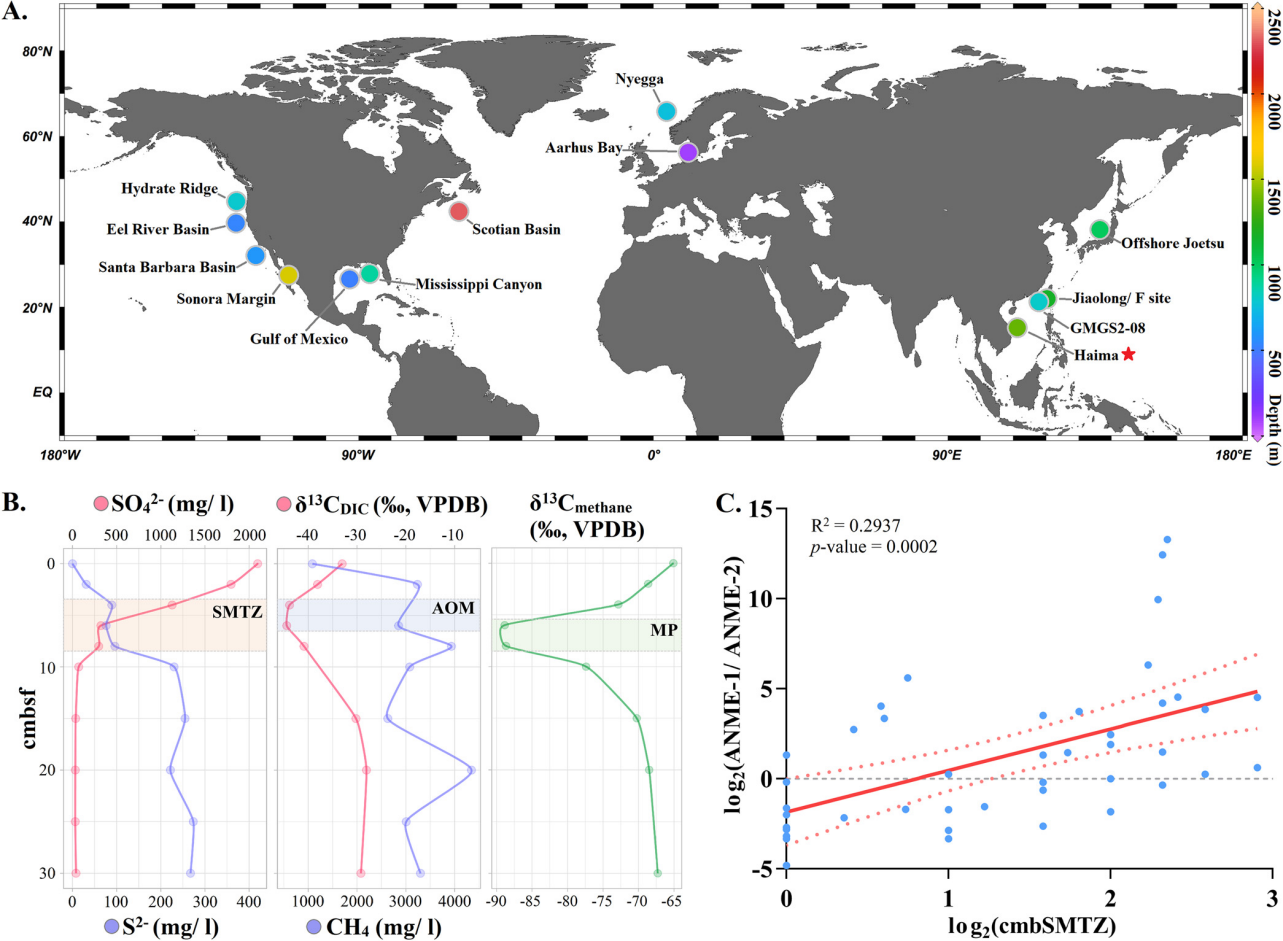
(A) Global methane seep ecosystems in which environmental contexts and prokaryotic community structures were reported over the last 2 decades. The sediment samples used in this study were collected from Haima methane seep (marked by a red star). Information on the methane seeps reviewed in this figure is available in Table S1. (B) Vertical profiles of environmental factors in sediment from the Haima methane seep, including sulfate (SO_4_^2−^), sulfide (S^2−^), methane (CH_4_), and δ^13^C values of DIC (δ^13^C_DIC_) and methane (δ^13^C_methane_); (C) linear regression between log_2_ (ANME-1 abundance/ANME-2 abundance) and log_2_ (cmbSMTZ). AOM, anaerobic oxidation of methane; MP, methane production; SMTZ, sulfate-methane transition zone; ANME, anaerobic methanotrophic archaea; cmbSMTZ, centimeters below the top of the SMTZ.

We reviewed global methane seep ecosystems in which environmental contexts and prokaryotic community structures had been reported over the last 2 decades (Fig. 1A; see Fig. S1 and Table S1 in the supplemental material). A typical environmental profile was identified in nearly all reported methane seep ecosystems, including the Haima methane seep in this study, where a high sulfate concentration characterized the top SMTZ, while deep sediment layers below the SMTZ were sulfate depleted but rich in sulfide and methane (Fig. 1B). A highly negative δ^13^C value of dissolved inorganic carbon (DIC) was detected in the SMTZ, indicating that biogenic CO_2_ was actively produced via AOM in this zone. Intriguingly, A highly negative δ^13^C value of methane was also detected in deep SMTZ, indicating that the ^13^C-depleted DIC produced by AOM is recycled back to methane with preferential use of [^12^C]CO_2_ during methane production (7, 8). The ^13^C depletion of DIC and methane in SMTZ can be interpreted as evidence for the intertwined anaerobic oxidation and production of methane (7). Although we did not measure oxygen concentration, the high sulfide concentration indicated that environments in deep layers are reductive and oxygen depleted, and oxygen in seawater is only likely to penetrate top sediments via molecular diffusion (5). In addition to the environmental profile, we also observed in a typical distributional profile in global methane seeps that ANME-2 archaea are more prevalent than ANME-1 archaea in shallow layers, while ANME-1 archaea are more prevalent in deep layers (Fig. S1).

10.1128/msystems.01179-22.1 FIG S1 (A) Bar plot of the relative abundance of ANME archaea among all archaea (stations colored in green) or *mcr* gene-containing microorganisms (stations colored in yellow) in different methane seep sites. The pink area indicates the SMTZ. (B) Bar plot of the qPCR abundance of each ANME clade in different methane seep sites. Information on the methane seeps reviewed in this figure is available in Table S1. ANME, anaerobic methanotrophic archaea; SMTZ, sulfate-methane transition zone. Download FIG S1, TIF file, 5.2 MB. Copyright © 2023 Chen et al. 2023 Chen et al. https://creativecommons.org/licenses/by/4.0/ This content is distributed under the terms of the Creative Commons Attribution 4.0 International license.

10.1128/msystems.01179-22.2 TABLE S1 Information of the methane seeps with ANME archaeal distribution pattern reported over the last two decades. ANME, anaerobic methanotrophic archaea. Download Table S1, DOCX file, 0.02 MB. Copyright © 2023 Chen et al. 2023 Chen et al. https://creativecommons.org/licenses/by/4.0/ This content is distributed under the terms of the Creative Commons Attribution 4.0 International license.

Although these two profiles are universal in global methane seeps, the depth and thickness of the SMTZ and the relative abundance of ANME archaeal clades vary significantly in different methane seep ecosystems. To normalize the variations, we used the logarithmic value of depth below the top layer of the SMTZ as an indicator of environmental factors and the logarithmic value of the abundance ratio of ANME-1 to ANME-2 archaea as an indicator of ANME archaeal communities. Only samples in which clades ANME-1 and ANME-2 coexisted were analyzed. Linear regression was then implemented using these two indicators. The results showed that the shift from communities where ANME-2 predominated in shallow sulfate-rich layers to communities in which ANME-1 predominated in deep sulfate-depleted layers is statistically supported in global methane seeps (*R*^2^ = 0.29; *P* < 0.01) (Fig. 1C).

### Genomic potential of ANME archaea in methane metabolism.

We went through all of the reactants of AOM process and controlling stressors of ANME archaea ever reported to identify the potential driving factors of the niche partitioning of ANME archaea in methane seeps. The main factor controlling AOM rates and the growth of AOM consortia is the availability of methane (the electron donor) (6) and electron acceptors (1). So far, three types of electron acceptors have been reported to couple to AOM, including different sulfur compounds (15–18), metal (19), and nitrate/nitrite (20). Apart from the reactants of AOM, other factors reported to be critical in shaping ANME archaeal distributions include sulfide, oxygen, temperature, salinity, and pH value (5, 21). However, temperature, salinity, and pH value may not lead to their niche partitioning in seeps because their variations in methane seep sediments are mild, which is within the optimum of ANME archaeal communities (5, 22–24). In contrast, oxygen and sulfide are likely to be critical in controlling the niche partitioning. ANME-1 archaea were reported to be more oxygen sensitive than ANME-2 archaea (12, 25), while ANME-2 archaea were more sensitive to hydrogen sulfide (26). Since the decrease of oxygen and increase of sulfide concentrations from top to deep SMTZ layers were frequently observed (5), the higher oxygen concentration in top sediments compared with deep layers may be a key factor inhibiting the growth of ANME-1 archaea, while the high sulfide concentration in deep layers can be a key factor restricting the population of ANME-2 archaea.

**FIG 2:**
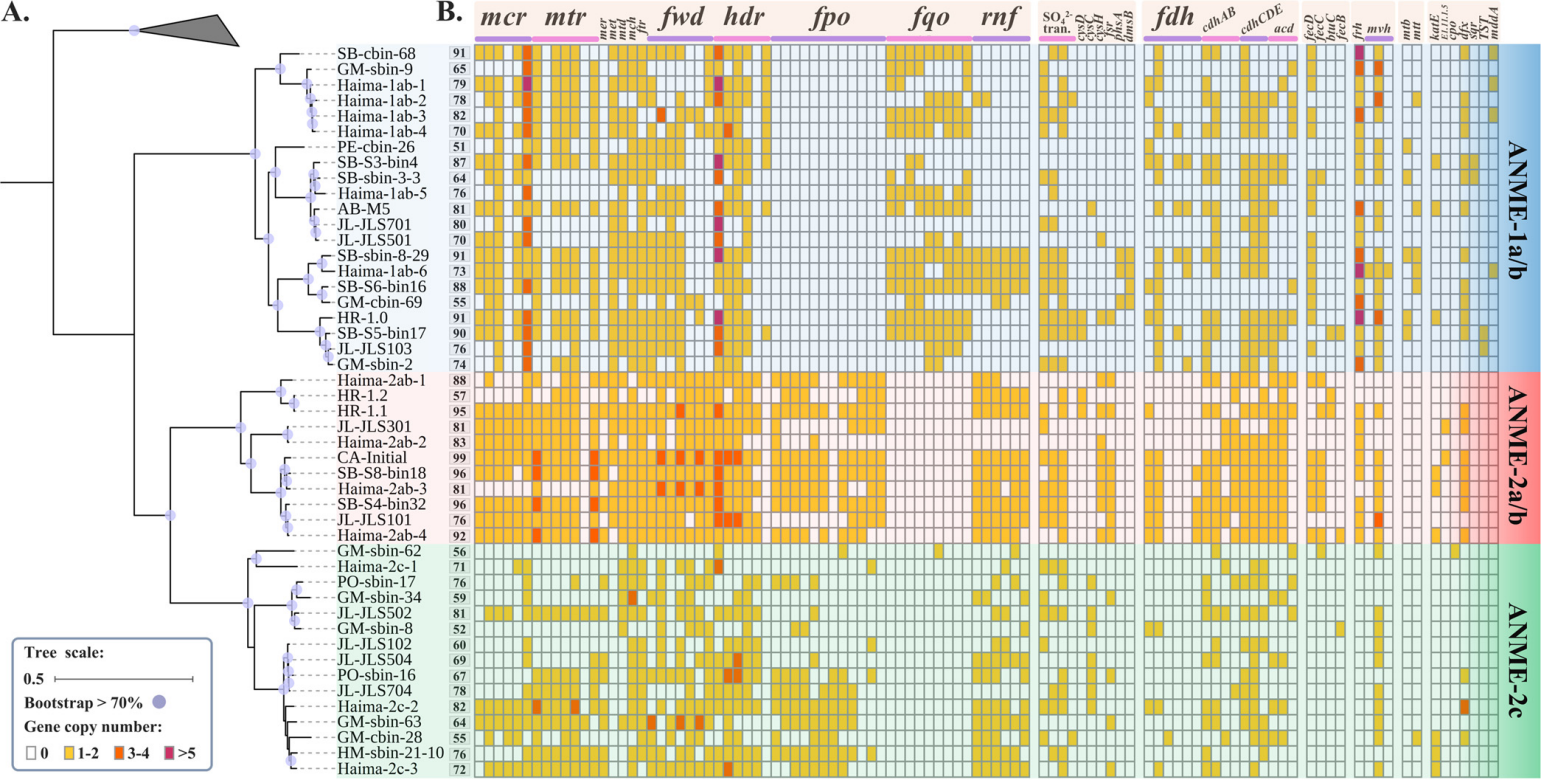
(A) Maximum likelihood tree based on a multiple-sequence alignment of archaeal single-copy marker proteins. This tree was constructed in IQ-TREE with 1,000 ultrafast bootstraps using the JTT+F+G4 model selected by ProtTest. Genomic information of ANME MAGs is listed in Table S2. (B) The heat map shows the completeness of each of the ANME archaeal MAGs and the copy number of genes in each genome. Detailed annotation results are available in Table S3. SO_4_^2−^ tran., SO_4_^2−^ transporters; ANME, anaerobic methanotrophic archaea; MAG, metagenome-assembled genome.

To investigate the genomic mechanisms of how the driving factors mentioned above lead to ANME archaeal niche partitioning in seep sediments, 63 ANME archaeal MAGs from global methane seeps were compared in this study (Table S2). Thirteen were recovered from the Haima methane seep, and the others were collected from publicly available databases. After dereplication, 47 MAGs of medium quality (completeness of >50% and contamination of <10%) to high quality (completeness of >90% and contamination of <5%) (27) were retained and used to investigate the relationship between ANME archaeal genomic features and the potential driving factors, including methane, sulfate, metal, nitrate/nitrite, oxygen, and sulfide. Taxonomic information was assigned to MAGs based on archaeal single-copy marker genes (Fig. 2A), showing that seep ANME archaeal MAGs were distinctly clustered into three clades, including ANME-1a/b (*n* = 21), ANME-2a/b (*n* = 11), and ANME-2c (*n* = 15).

10.1128/msystems.01179-22.3 TABLE S2 Genomic information of the 63 ANME archaeal MAGs used in this study. ANME, anaerobic methanotrophic archaea; MAG, metagenome-assembled genome. Download Table S2, XLSX file, 0.01 MB. Copyright © 2023 Chen et al. 2023 Chen et al. https://creativecommons.org/licenses/by/4.0/ This content is distributed under the terms of the Creative Commons Attribution 4.0 International license.

10.1128/msystems.01179-22.4 TABLE S3 Annotation results and the copy number of genes in ANME archaeal MAGs. ANME, anaerobic methanotrophic archaea; MAG, metagenome-assembled genome. Download Table S3, XLSX file, 0.03 MB. Copyright © 2023 Chen et al. 2023 Chen et al. https://creativecommons.org/licenses/by/4.0/ This content is distributed under the terms of the Creative Commons Attribution 4.0 International license.

Both ANME-1 and ANME-2 archaea employed genes necessary to produce enzymes that anaerobically convert methane to CO_2_, albeit ANME-1 and ANME-2 archaea used different enzymes to produce the interconversion between methyl-H_4_MPT and methylene-H_4_MPT (ANME-1 archaea carried only a gene that encoded methylenetetrahydrofolate reductase [*met*], while ANME-2 archaea carried both the *met* gene and a gene encoding N^5^,N^10^-methylene tetrahydromethanopterin reductase [*mer*]) and the reduction of F_420_H_2_ to F_420_ (ANME-1 archaea carried a gene encoding F_420_H_2_:quinone oxidoreductase [*fqo*], while ANME-2 archaea carried a gene encoding F_420_H_2_:methanophenazine oxidoreductase [*fpo*]) (Fig. 2B; Table S3). So far, no studies have investigated if *met* and *mer* have different catalytic efficiencies, and neither have those of *fqo* and *fpo* been investigated. Therefore, we cannot suggest whether these genomic variations lead to a different performance in AOM among ANME archaeal clades. In fact, the relationship between methane concentration and AOM in different ANME archaeal clades is debatable. Yanagawa et al. (2011) and Girguis et al. (2005) demonstrated that high methane flux in active sites benefits ANME-1 more than ANME-2 archaea (15, 28). However, Nauhaus et al. (2005) found that the community dominated by ANME-2 showed significantly higher cell-specific AOM rates when sufficient methane was provided (22). Considering AOM is controlled by both electron donors and acceptors, the inconsistent results from different studies suggest that methane can only partially define the niche partitioning of ANME archaea in methane seep sediments, and the electron acceptors in AOM must be investigated.

### ANME-2 archaea perform better in direct interspecies electron transfer and benefit more from electron acceptors in AOM.

**FIG 3:**
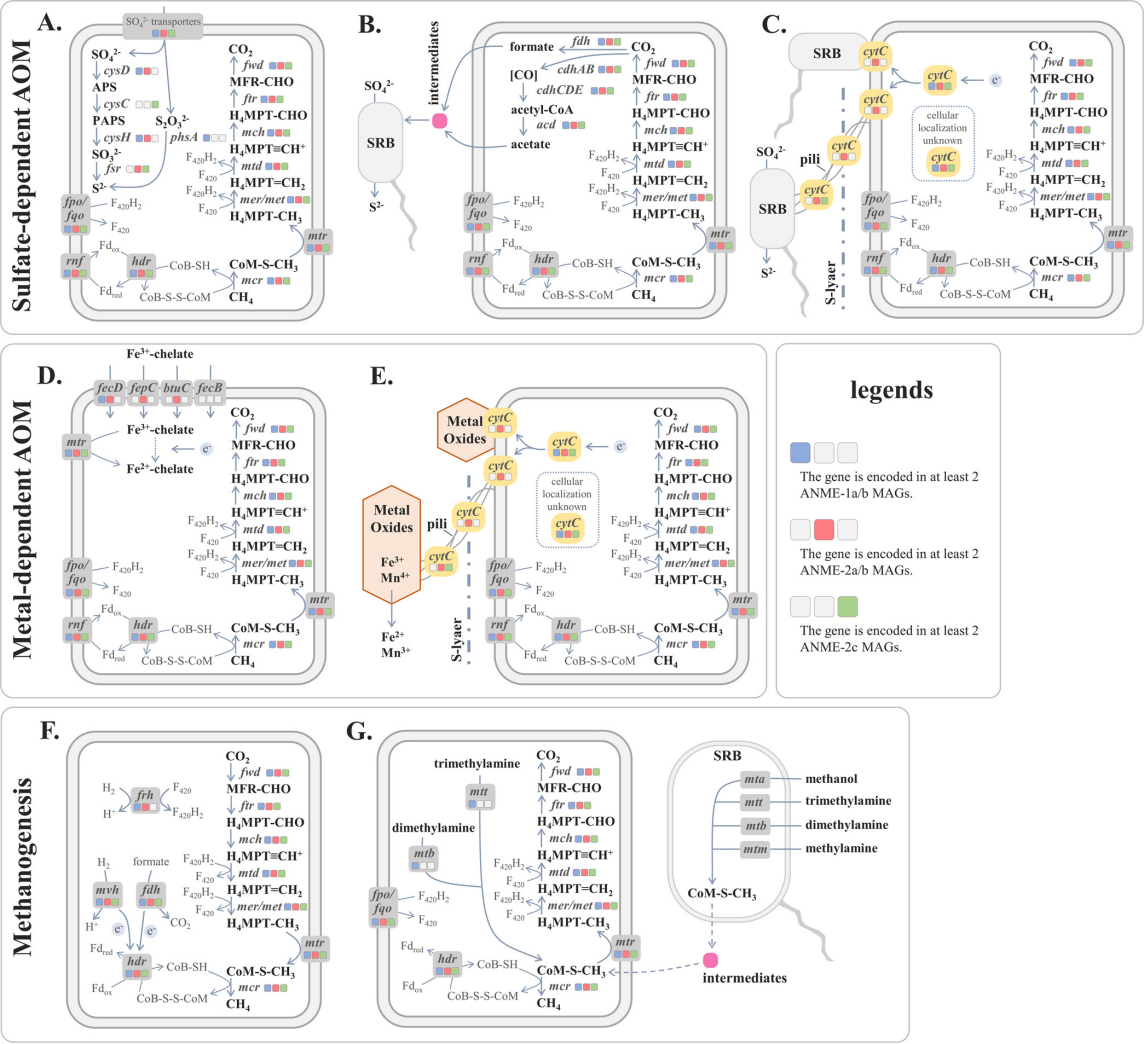
(A) Nonsyntrophic ANME archaea perform sulfate-dependent AOM solely. (B) Syntrophic ANME archaea perform sulfate-dependent AOM in consortia with SRB mediated by intermediate compounds. (C) Syntrophic ANME archaea perform sulfate-dependent AOM in consortia with SRB mediated by DIET. (D) Nonsyntrophic ANME archaea perform metal-dependent AOM using chelate metal ions. (E) Nonsyntrophic ANME archaea perform metal-dependent AOM via direct electron transfer to metal nanoparticulate by contact and nanowires. (F) ANME archaea perform hydrogenotrophic methanogenesis solely. (G) ANME archaea perform methylotrophic methanogenesis solely and/or in consortia with SRB mediated by intermediate compounds. Detailed annotation results are available in Table S3. cytC, *c*-type cytochromes; ANME, anaerobic methanotrophic archaea; SRB, sulfate reduction bacteria; DIET, direct interspecies electron transfer.

AOM has been reported to couple to the reduction of sulfate, metal, and nitrate/nitrite. To complete the AOM process, ANME archaea can either exhibit syntrophic associations with bacterial partners or perform it solely (6). We first investigate the potential of ANME archaea to perform AOM-SR process solely (Fig. 3A). Our results showed that only genes involved in assimilatory but not dissimilatory SR were detected in ANME archaeal MAGs (Fig. 2B). Putative sulfate transporters were identified in nearly all MAGs, but only 2 in 21 (9.52%) ANME-1a/b, 2 in 11 (18.18%) ANME-2a/b, and 0 in 15 (0%) ANME-2c archaea encoded heterodimeric sulfate adenylyltransferase (*cysD*), which catalyzes the first step of SR, suggesting that most ANME archaea cannot perform complete SR started from inorganic sulfate. Notably, we found that the coenzyme F_420_-dependent sulfite reductase (*fsr*), which reduces sulfite (sulfur oxidation state of +4) to sulfide (sulfur oxidation state of −2), was encoded in more ANME-2 (46.15% [12 in 26]) than ANME-1 (4.76% [1 in 21]) strains. Since the high expression of *fsr* in ANME archaea has been verified based on previous metatranscriptomic and proteomic analyses (29, 30), our results suggest that more ANME-2 strains can take advantage of sulfite as electron acceptors compared with ANME-1 strains. Although it has been reported that ANME archaea alone can perform SR (29, 30), 48.94% of the ANME archaeal MAGs (17 in 21 ANME-1a/b, 1 in 11 ANME-2a/b, and 5 in 15 ANME-2c) did not carry any gene involved in the assimilatory SR pathway. To assess the statistical likelihoods for the presence or absence of an assimilatory SR pathway in ANME archaeal MAGs, we use MetaPOAP to calculate the false-negative estimate, showing that 15 in 21 (71.42%) ANME-1a/b, 6 in 11 (54.55%) ANME-2a/b, and 5 in 15 (33.33%) ANME-2c MAGs might miss some marker genes in SR and only contain a partial SR pathway (Table S4). In general, the genetic potential of SR is diverse in different ANME archaeal clades, whereby ANME-2 archaea encode more SR-related genes than ANME-1 archaea, and the ability to perform SR alone is not widespread in ANME archaeal strains.

10.1128/msystems.01179-22.5 TABLE S4 Statistical likelihoods of the presence or absence of sulfate reduction pathways in ANME archaeal MAGs assessed by MetaPOAP using four marker genes, including *cysD*, *cysC*, *cysH*, and *fsr*. ANME, anaerobic methanotrophic archaea; MAG, metagenome-assembled genome. Download Table S4, XLSX file, 0.01 MB. Copyright © 2023 Chen et al. 2023 Chen et al. https://creativecommons.org/licenses/by/4.0/ This content is distributed under the terms of the Creative Commons Attribution 4.0 International license.

The frequently reported co-occurrence of ANME archaea and SRB in AOM communities, the tight physical association between ANME archaea and SRB observed through FISH analysis (31–33), and the isotopic signatures in lipid biomarkers of ANME archaea and SRB (34, 35) indicate a syntrophic relationship between the two. Two models have been proposed to describe their relationship. First, the electrons produced through the AOM process were transferred from ANME archaea to SRB through intermediate compounds (Fig. 3B), such as formate, acetate, hydrogen, and methanol. Second, ANME archaea directly transfer electrons to SRB through conductive cell-to-cell connections (nanowires) (Fig. 3C). For the first model, formate and acetate are the most potential intermediates (6). Our results show that genes involved in the synthesis of formate (formate dehydrogenase [*fdh*]) and acetate (reversible CO dehydrogenase/acetyl-coenzyme A [CoA] synthetase [*cdhAB*], AMP-forming acetyl-CoA synthetase [*cdhCDE*], and ADP-forming acetate-CoA ligase [*acd*]) were carried in all ANME archaeal clades (Fig. 3C and Fig. 2B), indicating both ANME-1 and ANME-2 archaea have genetic potential to synthesize the intermediate compounds and can efficiently reduce sulfate in cooperation with SRB. However, the addition of acetate or formate in AOM enrichment incubation systems did not lead to the decoupling of AOM and SR or any change in SR rate, whereas the reaction should be shifted to lower AOM rates upon the addition of intermediates if the first model is accurate (22, 36, 37). Therefore, whether these compounds are AOM electron shuttles remains to be confirmed.

**FIG 4:**
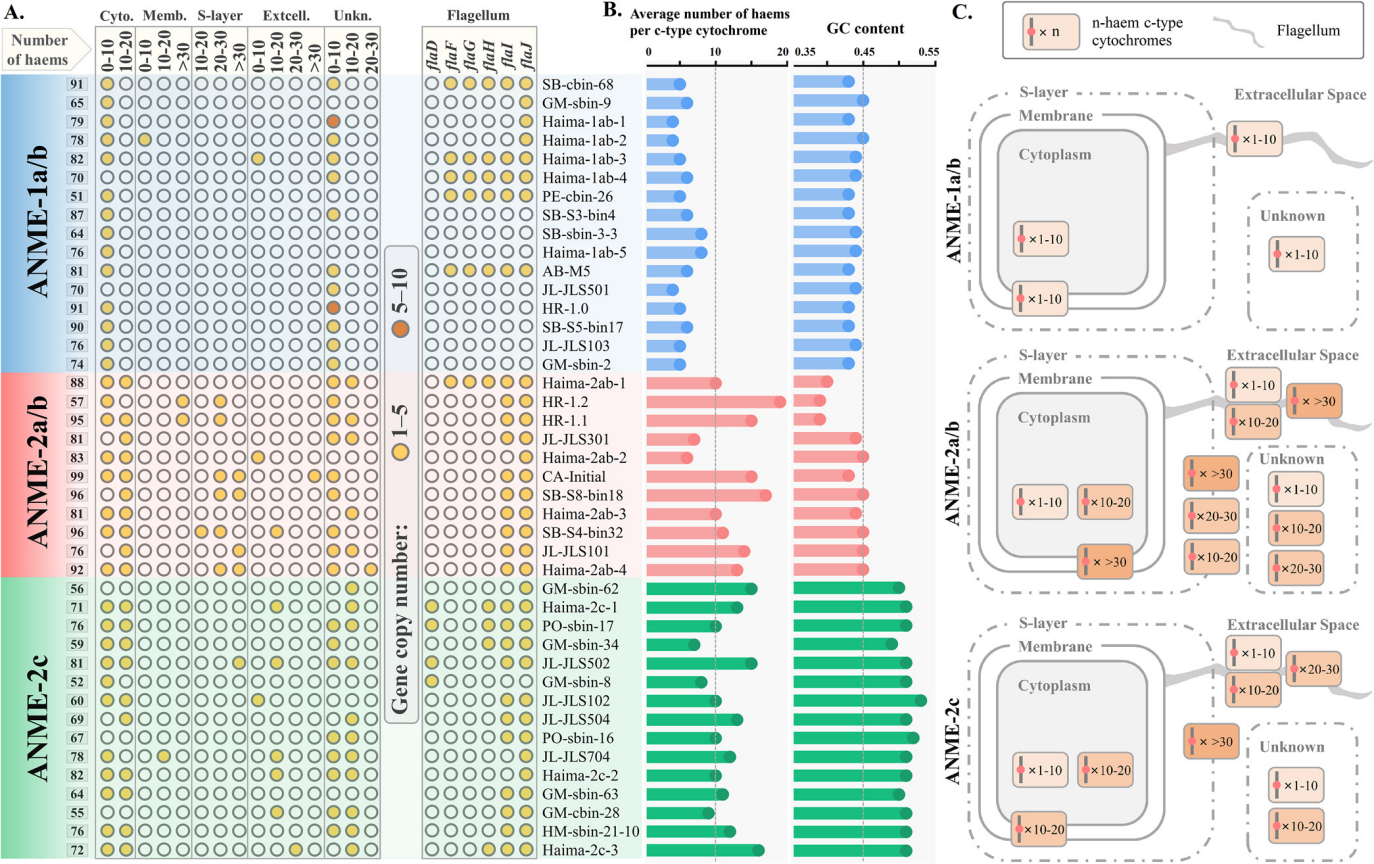
(A) Copy number of *c*-type cytochrome at different subcellular localizations with a different number of hemes in each ANME archaeal genome and the copy number of flagellar proteins. Only ANME archaeal strains encoding *c*-type cytochrome are listed in this figure. The predicted subcellular localization of *c*-type cytochrome is indicated by the following abbreviations: cyto., cytoplasm; memb., membrane; S-layer, surface layer; extcell., extracellular space; unkn., unknown. (B) Average number of hemes per *c*-type cytochrome and GC content of each genome; (C) model of the *c*-type cytochrome with a different number of hemes in each ANME archaeal clade. For further details used in this figure, see Table S3 and Table S5. ANME, anaerobic methanotrophic archaea.

**FIG 5:**
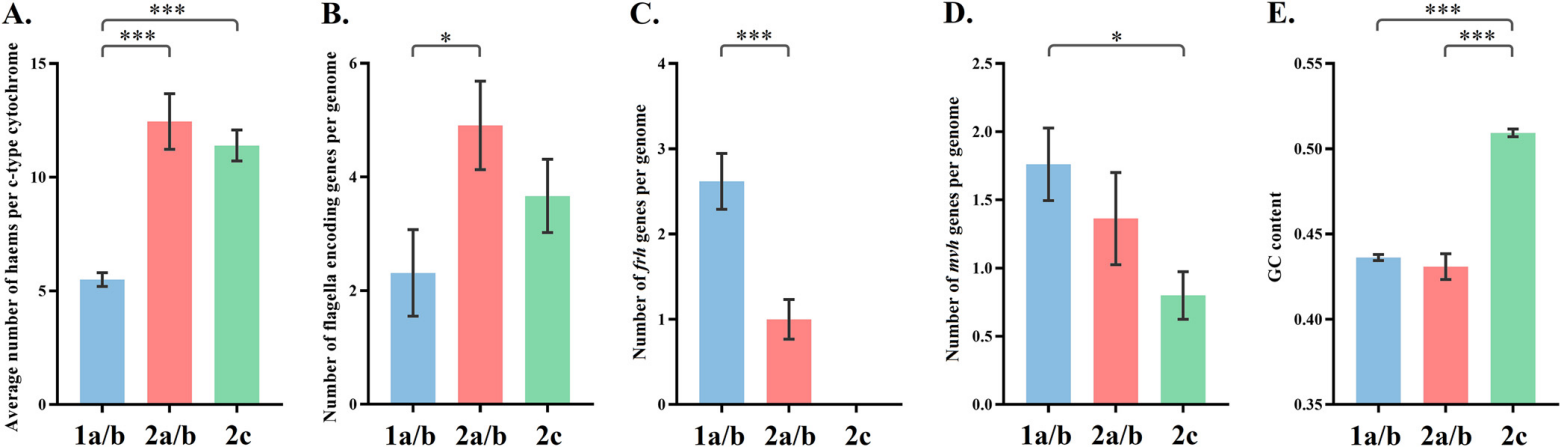
(A) Average number of hemes per *c*-type cytochrome in different ANME clades; (B) number of flagellum-encoding genes per genome in different ANME archaeal clades; (C) number of *frh* genes per genome in different ANME archaeal clades; (D) number of *mvh* genes per genome in different ANME archaeal clades; (E) GC content of each genome in different ANME archaeal clades. Results of statistical analyses are available in Table S8. Error bars represent standard deviations. *, *P* < 0.05; **, *P* < 0.01; ***, *P* < 0.001. ANME, anaerobic methanotrophic archaea.

In contrast, the second model has been recently reported to best fit the empirical data (38, 39). Although the process of direct interspecies electron transfer (DIET) is not fully understood, two groups of proteins were reported to be essential to DIET, including flagellar proteins, which create physical contact among cells, and outer membrane *c*-type cytochromes, which conduct the electron transfer (40, 41). Intriguingly, we identified significant genomic differences between the ANME-1 and ANME-2 archaea regarding these proteins. First, genes encoding flagella and *c*-type cytochromes were identified in all ANME-2 strains, while only 47.62% (10 in 21) of ANME-1 strains contained genes that encoded flagella and 76.19% (16 in 21) of ANME-1 strains contained genes that encoded *c*-type cytochrome proteins (Fig. 4A; Table S3 and Table S5). Second, the average number of hemes per *c*-type cytochrome and the copy number of flagellum-encoding genes, which are positively related to the efficiency of electron transfer and stability of cytochromes (42), were significantly higher in ANME-2 than ANME-1 strains (Fig. 4B and Fig. 5A and B). Moreover, although *c*-type cytochromes were encoded in some ANME-1 strains, most of them were only localized in the cytoplasm, while many multiheme (>10 hemes) *c*-type cytochromes in ANME-2 strains had a putative localization on outer membrane areas, including the S-layer and extracellular space (Fig. 4C). Considering the observations that ANME-1 archaea often occur as single cells, while ANME-2 archaea often form coccoid consortia with SRB in marine sediments (1, 6, 33), our results provide genomic evidence to support that ANME-2 strains can better perform DIET and exhibit a stronger association with their syntropic bacteria, and thus, they may get more benefits from sulfate than ANME-1 archaea.

10.1128/msystems.01179-22.6 TABLE S5 Annotation results and subcellular localization predictions of *c*-type cytochrome in each ANME MAGs. ANME, anaerobic methanotrophic; MAG, metagenome-assembled genome. Download Table S5, XLSX file, 0.02 MB. Copyright © 2023 Chen et al. 2023 Chen et al. https://creativecommons.org/licenses/by/4.0/ This content is distributed under the terms of the Creative Commons Attribution 4.0 International license.

10.1128/msystems.01179-22.9 TABLE S8 (A) Shapiro-Wilk test results show if each group's numeric data were normally distributed; (B) evaluation of group differences. A nonparametric test (Wilcoxon’s test [wilcox.test]) was implemented to evaluate the differences among groups with abnormal distribution, and a *t* test (t.test) was implemented to evaluate the differences among groups with normal distribution. NA, not applicable; ANME, anaerobic methanotrophic archaea. Download Table S8, DOCX file, 0.02 MB. Copyright © 2023 Chen et al. 2023 Chen et al. https://creativecommons.org/licenses/by/4.0/ This content is distributed under the terms of the Creative Commons Attribution 4.0 International license.

Both soluble and nanoparticulate metals support methane-oxidizing activity (19, 43). Free Fe^3+^ and Mn^4+^ ions in the anoxic sediments readily form precipitates with very low solubility. However, many microbes can secrete intermediates such as citrate or specially synthesized siderophores that chelate metal ions and make them accessible for cellular uptake (44). There are three reported modes for metal-dependent AOM. First, ANME archaea oxidize methane and transfer electrons directly to chelate metal ions (Fig. 3D). Second, direct electron transfer to metal nanoparticulate can occur by contact and/or nanowires (Fig. 3E). Third, ANME archaea can be partnered with metal-reducing bacteria (MRB) to perform AOM, like ANME archaeon-SRB consortia. Annotation results from the TransportDB2 database showed that four Fe^3+^-chelate transporters were encoded in ANME archaeal MAGs (Fig. 2B and Fig. 3D). One Fe^3+^ citrate transporter gene (*fecD*) was carried in both ANME-1a/b and ANME-2a/b strains, while three Fe^3+^ siderophore transporter genes (*fepC*, *btuC*, and *fecB*) were carried in only one ANME-1a/b strain but carried in most ANME-2a/b strains. This result indicated that ANME-2a/b strains might have more advantages from Fe^3+^ siderophore iron. For the second and third models, DIET still plays crucial roles in metal-dependent AOM, and thus ANME-2 archaea can perform them better than ANME-1 archaea.

Denitrifying AOM (DAMO) that couples to nitrate and nitrite was frequently reported in freshwater sediments (45, 46) but was first reported in methane seep sediment in 2014 (20). We measured the concentrations of nitrate and nitrite in the Haima methane seep and found that nitrate can only be detected at the surface layer (0 to 2 cm), while nitrite cannot be detected in seep sediments (Table S6). This environmental profile indicated that DAMO might only happen in shallow sediments. Like AOM-SR, DAMO can be performed by ANME archaea solely or in consortia with partner bacteria of the *Desulfobacteraceae*, which perform the denitrification and accept the electrons from the AOM process. However, genes involved in denitrification (i.e., the nitrate reductase gene *nar* and nitrite reductase gene *nir*), were absent in all ANME archaeal MAGs recovered from methane seep sediments, indicating the association between ANME archaea and *Desulfobacteraceae* is obligated for DAMO. Although a distinct ANME archaeal strain (ANME-2d) recovered from freshwater sediment was reported to perform DAMO solely (47), this strain was not successfully recovered from any seep sediments. Therefore, DAMO in methane seeps also rely on DIET, and thus, ANME-2 archaea are likely to perform DAMO better than ANME-1 archaea.

10.1128/msystems.01179-22.7 TABLE S6 Concentrations of nitrate and nitrite in Haima methane seep sediments. cmbsf, centimeters below the seafloor. Download Table S6, DOCX file, 0.02 MB. Copyright © 2023 Chen et al. 2023 Chen et al. https://creativecommons.org/licenses/by/4.0/ This content is distributed under the terms of the Creative Commons Attribution 4.0 International license.

Among the three AOM processes in methane seep sediments, ANME-2 archaea exhibit genomic potential to perform them better than ANME-1 archaea. Although a pure culture of ANME archaea is so far not available, the AOM rates in enrichment cultures of different clades of ANME archaea have been reported in some studies. High AOM rates (>200 μmol g dry weight^−1^ day^−1^) in ANME-2 enrichment cultures were reported in at least two separate studies, while the highest AOM rate ever reported in ANME-1 enrichment cultures was 13.5 μmol g dry weight^−1^ day^−1^, which is ~20 times lower than in ANME-2 enrichment cultures (reviewed by Bhattarai et al. [2019] in reference 6). The results of *in vitro* incubations are consistent with our molecular prediction. Therefore, we hypothesize that ANME-2 archaea have genomic potential to perform AOM better, making ANME-2 more prevalent than ANME-1 in the shallow SMTZ layer.

### ANME-1 archaea can perform methanogenesis better than ANME-2 archaea.

The shift from predominant AOM in the shallow SMTZ to predominant MP in deep sediments has been identified frequently in different methane seeps through the systematic discrepancy between AOM and SR rates, and ^13^C isotope signatures of DIC and methane (7–9, 48–50). Consistently, an active MP zone in the deep SMTZ has been observed in the Haima methane seep in this study (Fig. 1B). Since SRB have a lower half-saturation constant for hydrogen and acetate than methanogens, they can outcompete methanogens in shallow methane seep sediments where sulfate is not a limiting factor (51, 52). Therefore, the shifting to reductive conditions and depletion of sulfate in deep methane seep sediment layers may lead to an MP-favored environment.

ANME archaea had been assumed to be obligate methanotrophs until Lloyd et al. (2011) found environmental evidence for net methane production in a community where clade ANME-1 predominated (49). After that, more studies reported that both ANME-1 and ANME-2 archaea could possess methanogenic capabilities (8, 15, 18, 53, 54). ANME archaea perform AOM via a reverse-methanogenesis pathway (6, 55) and possess complete enzymes involved in the interconversion between CH_4_ and CO_2_. So far, three types of methanogenesis are widely employed in methane-producing archaea: hydrogenotrophic, methylotrophic, and acetoclastic (56, 57). Previous studies reported that acetoclastic methanogenesis could not be detected in ANME archaeal communities (53). Therefore, acetoclastic methanogenesis was excluded from potential MP in ANME archaea. Since methylotrophic methanogenesis was successfully observed in ANME archaeal communities (53) and no empirical data eliminated the potential of hydrogenotrophic methanogenesis, we focused on only the genomic potential of methylotrophic and hydrogenotrophic methanogenesis in ANME archaea.

In hydrogenotrophic methanogenesis, hydrogen is utilized as an electron donor for the reduction of carbon dioxide to methane (Fig. 3F). Two main hydrogenases are used for the oxidation of dihydrogen: the coenzyme F_420_ hydrogenase subunit beta (*frh*), which reduces the methanogenic cofactor F_420_ to F_420_H_2_, and the soluble Mvh hydrogenase (*mvh*), which forms a complex with heterodisulfide reductase (*hdr*) and couples the oxidation of dihydrogen to the reduction of ferredoxin and the heterodisulfide CoM-S-S-CoB (58). The *frh* gene was detected in ANME-1a/b and ANME-2a/b but not in ANME-2c strains, while *mvh* was detected in all ANME archaeal clades (Fig. 2B). Remarkably, the copy number of *frh* gene in ANME-1a/b strains was significantly higher than that in ANME-2a/b strains (Fig. 5C), and the copy number of the *mvh* gene in ANME-1a/b strains was also higher than other those in ANME-2 strains (Fig. 5D), indicating ANME-1 can perform hydrogenotrophic methanogenesis better than ANME-2 strains.

In methylotrophic methanogenesis, methane can be derived from the reduction of CO_2_ from different methyl group substrates (57). We identified two enzymes involved in methylotrophic methanogenesis in ANME archaeal MAGs: one is dimethylamine corrinoid protein (*mtb*), whose substrate is dimethylamine, and the other one is trimethylamine-corrinoid protein Co-methyltransferase (*mtt*), whose substrate is trimethylamine (Fig. 3G and Fig. 2B). ANME-1 clade archaea carried both methylotrophic genes, while ANME-2a/b archaea did not carry any of them, and only one ANME-2c strain carried the *mtt* gene (Fig. 2B). These results suggested that ANME-1 can perform methylotrophic methanogenesis with two different methyl group substrates solely, while ANME-2 may lack this capability. However, this genomic prediction is inconsistent with previous incubation experiments in which methylotrophic methanogenesis using methanol was observed in both ANME-1 and ANME-2 enriched cultures (53). One possible explanation is that the methylotrophic methanogenesis in an ANME archaea enriched culture could be performed by the consortia of ANME archaea and SRB. To examine the possibility, we investigated the genomic features of seven SEEP-SRB1 strains. Our results showed that SRB carried genes involved in the oxidation of methanol (*mta*), trimethylamine (*mtt*), dimethylamine (*mtb*), and methylamine (*mtm*), but did not carry the *mcr* gene to further convert methyl-CoM to methane (Table S7). These results supported our assumption and indicated that methyl-CoM might be a potential intermediate between ANME archaea and SRB for methylotrophic methanogenesis (Fig. 3G). In general, ANME-1 strains have the genetic potential to perform methylotrophic and hydrogenotrophic methanogenesis better than ANME-2 strains, facilitating their predominance in deep sediment layers.

10.1128/msystems.01179-22.8 TABLE S7 KEGG annotation results and the copy number of genes in SRB MAGs. KEGG, Kyoto Encyclopedia of Genes and Genomes; SRB, sulfate reduction bacteria. MAG, metagenome-assembled genome. Download Table S7, XLSX file, 0.1 MB. Copyright © 2023 Chen et al. 2023 Chen et al. https://creativecommons.org/licenses/by/4.0/ This content is distributed under the terms of the Creative Commons Attribution 4.0 International license.

### ANME-2 archaea are more tolerant to oxygen, while ANME-1 archaea have higher resistance to sulfide toxicity.

*In vitro* AOM activity is inhibited in the presence of oxygen (59). Strict anaerobes, such as SRB, cannot grow at pO_2_ levels greater than 0.5% (60). Therefore, oxygen concentration plays a vital role in shaping AOM communities. Three proteins that are essential in oxygen resistance, including peroxidase, catalase, and superoxide reductase (SOR) (61, 62), were identified in ANME archaeal MAGs (Fig. 2B). Our results show that all ANME archaeal clades have the genetic potential to synthesize SOR and catalase, while peroxidase was only identified in ANME-2 strains. Peroxidase was reported to be employed only by aerobic and facultative anaerobes, and bacteria carrying genes that encode peroxidase have a significantly higher level of oxygen resistance than other bacteria without peroxidase (61). These results suggest that ANME-2 strains have the genetic potential to tolerate oxygen better than ANME-1 strains. Moreover, the average GC content of the ANME-2c strain (0.51) is significantly higher than those in the ANME-1a/b (0.44) and ANME-2a/b (0.43) strains (Fig. 5D). High GC content is selected by oxic environment and high rates of DNA damage because GC alleles fix at a higher rate than AT alleles (63), indicating that ANME-2c may tolerate oxygen better than other ANME archaeal clades. In addition to molecular strategies, aggregate formation may be one behavioral strategy enabling ANME-2 archaea to tolerate oxygen better than ANME-1 archaea. The associated SRB consortia are strict anaerobes but are often found in environments where oxic conditions can temporarily exist, and their aggregations occur rapidly under conditions with oxygen influx (64, 65). It has been confirmed that cell aggregates create oxygen gradients from outer to inner layers, in which oxygen can only penetrate to the depth of 1.5 μm into the aggregates (64). Therefore, oxygen can be depleted in the core of cell aggregates of 3 μm or larger. Considering that ANME-2 archaea often form cell aggregates covered by SRB, but ANME-1 archaea often occur as single cells or chains of cells, ANME-2 archaea may also benefit from this behavioral strategy and exhibit stronger oxygen resistance than ANME-1 archaea.

Hydrogen sulfide in sediments significantly determines toxicity to resident organisms through its ability to inhibit *c*-type cytochrome oxidase (66–68). The growth of SRB is inhibited under high concentrations of hydrogen sulfide (69). Therefore, the advantages of ANME-2 strains that derive from DIET could be negatively impacted. For molecular strategy, we identified three genes that are functional in sulfide detoxification in ANME archaeal MAGs, including the genes coding for sulfide:quinone oxidoreductase (*sqr*) (70), thiosulfate sulfurtransferase (*TST*), and methanethiol *S*-methyltransferase (*mddA*) (71). However, these genes were distinctively carried in ANME-1a/b strains, while none of them was carried in ANME-2 strains (Fig. 2B), indicating a higher sulfide resistance in ANME-1 than ANME-2 archaea.

### Conclusions.

**FIG 6:**
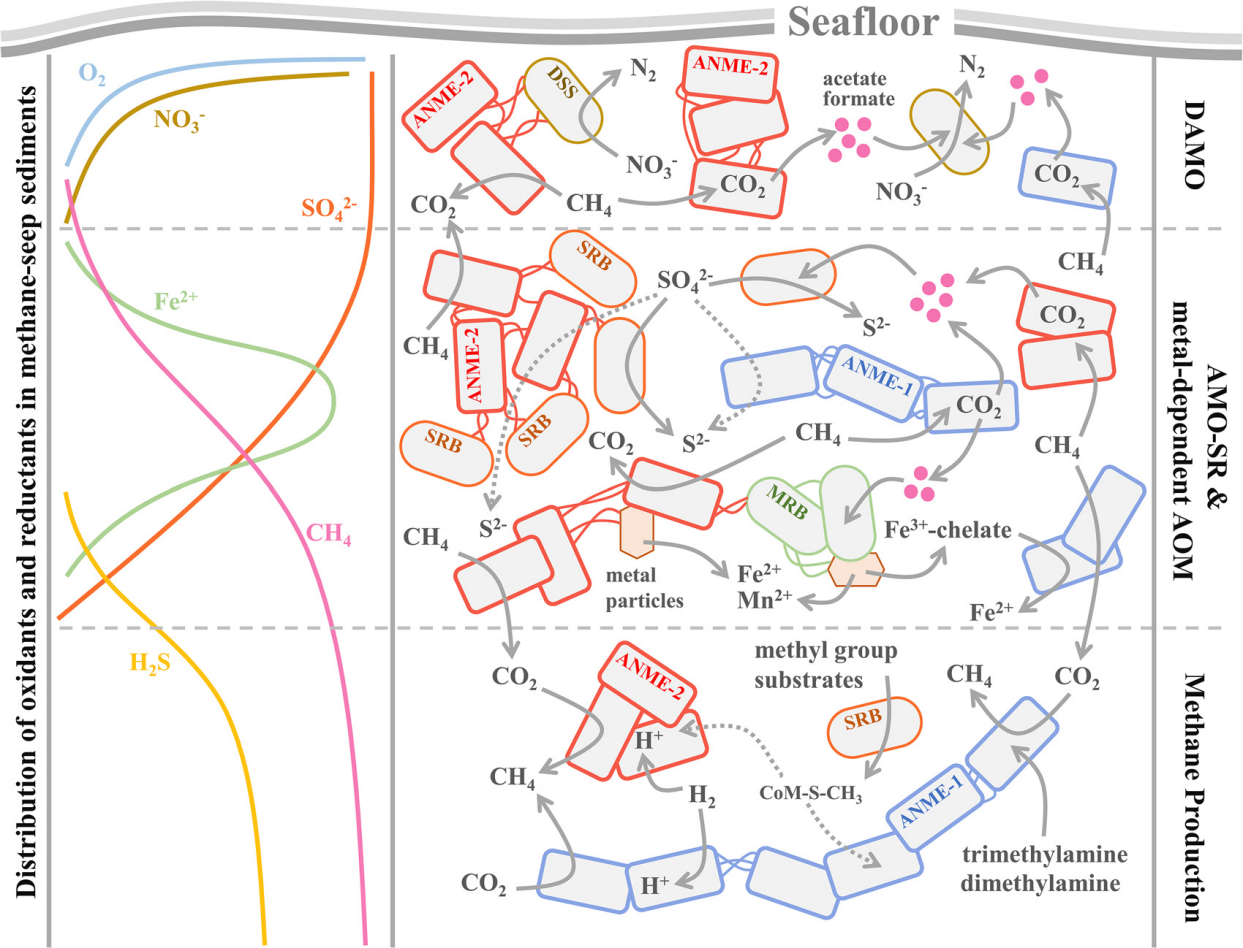
Proposed conceptual diagram of mechanisms underlying niche partitioning among ANME archaea in methane seep sediments. AMO, anaerobic oxidation of methane; DAMO, denitrifying anaerobic oxidation of methane; ANME, anaerobic methanotrophic archaea; DSS, *Desulfobacteraceae*; SRB, sulfate reduction bacteria; MRB, metal reduction bacteria.

ANME archaea play a crucial role in the global carbon cycle and climate change. Although their physiological responses to different environmental factors were investigated, the underlying genomic mechanisms are not fully understood. Through comparative genomic analyses, we demonstrate that the genomic potential of AOM and MP, as well as the adaptations to sulfide and oxygen, may jointly shape the distribution of ANME archaea in methane seep sediments. Our main findings are summarized in a conceptual diagram in Fig. 6. We found that, at the top of SMTZ, ANME-2 archaea can be more abundant than ANME-1 due to their better performance in the three types of AOM. For AOM-SR, ANME-2 archaea may benefit more from the high concentration of ambient sulfate than ANME-1 archaea because nearly half of the investigated ANME-2 strains carry genes responsible for the reduction of sulfur species, while most ANME-1 strains do not carry any SR-related genes. For metal-AOM, ANEM-2 archaea distinctively encode siderophore metal transporters (*fepC*, *btuC*, and *fecB*) supporting the uptake of extra chelate Fe^3+^ ions as electron acceptors. For DAMO, both ANME-1 and ANME-2 archaea in methane seeps cannot perform it without bacterial consortia. In general, ANEM-2 archaea have genetic potential to perform AOM solely better than ANME-1 archaea. Moreover, ANME-2 archaea carry genes that encode a significantly higher number of outer membrane multiheme *c*-type cytochromes and flagellar proteins than ANME-1 archaea, strengthening the association between ANME-2 archaea and bacterial consortia or metal particulates and facilitating the DIET among them. Additionally, the higher oxygen concentration in shallow sediments than in deep layers less inhibits the growth of ANME-2 than ANME-1 archaea because of the extra genes encoding peroxidase and high GC content in ANME-2 archaea, as well as the frequent formation of ANME-2 archaeon-SRB cell aggregates, make ANME-2 tolerate oxygen toxicity better than ANME-1 archaea. In contrast, ANME-1 archaea are more competitive than ANME-2 archaea in the deep sulfate-depleted MP zone. This is because they carry extra genes (*mtb* and *mtt*) for methylotrophic methanogenesis and a significantly higher number of *frh* and *mvh* genes for hydrogenotrophic methanogenesis. Additionally, ANME-1 archaea carry extra genes (*sqr*, *TST*, and *mddA*) involved in sulfide detoxification compared with ANME-2 archaea, resulting in a stronger sulfide resistance in ANME-1 archaea. Overall, this study reveals that genomic adaptation is a fundamental force in shaping the niche partitioning among ANME archaea in global methane-seep sediments.

## MATERIALS AND METHODS

### Data collection.

The vertical niche partitioning among ANME archaea in global methane seep sediments was identified based on the ANME archaeal community structures reported in 14 methane seeps (see Table S1 in the supplemental material). These sites were as follows: Eel River Basin (11), Gulf of Mexico (13), Hydrate Ridge (12), Santa Barbara Basin (72), offshore Joetsu (15), Nyegga G11 and CN03 (73, 74), Sonora Margin (75), Mississippi Canyon (76), Haima (16), Aarhus Bay (8), GMGS2-08 (17), Jiaolong F3 site (18), and Scotian Basin (77). A total of 50 ANME archaeal MAGs were collected from public databases (Table S2), including one from Eel River Basin (55), one from a Black Sea microbial mat (78), three from Hydrate Ridge (29), one from Aarhus Bay (8), five from the Gulf of Cadiz (79), 13 from the Jiaolong F3 site (80), 14 from the Scotian Basin (77, 81), one from the Eastern North Pacific, one from Haakon Mosby, two from Santa Monica Mounds, and eight from the Gulf of Mexico (81). SEEP-SRB1 MAGs were recovered from Hydrate Ridge and the Santa Monica Mounds by Skennerton et al. (2017) (82).

### Sampling and geochemical analyses.

Sediment samples used in this study were collected from Haima methane seep (water depth of ~1,400 m) (Fig. 1A) in the South China Sea during the cruise HYDZ6-202102 (R/V *Haiyangdizhi VI*, May 2021). Three push cores with a sediment depth of 30 to 70 cm were retrieved from active seep area using the remotely operated underwater vehicle (ROV) *Haima*. Porewater for geochemical analyses was collected using a Rhizon sampler (Rhizosphere Research Products, Wageningen, Netherlands) in a cold room at 4°C. After porewater collection, sediment cores were subsampled aseptically for metagenomic sequencing in 2- or 5-cm-thick layers. All sediment samples were frozen and kept at −20°C on board, transferred to the lab in dry ice, and stored at −80°C in the lab until further use. Concentrations of methane, sulfate, sulfide, and nitrate/nitrite were measured using an Agilent 6850 series II gas chromatography (GC) device (Agilent, Santa Clara, CA, USA), a Dionex ICS-1100 ion chromatography system (Thermo Fisher Scientific, Poway, CA, USA), a SmartChem200 wet chemistry analyzer (KPM Analytics, Westborough, MA, USA), and a San^++^ continuous flow analyzer (Skalar, Netherlands), respectively. The stable carbon isotopic composition of DIC and methane was measured on a Delta V Advantage mass spectrometer (Thermo Fisher Scientific, Poway, CA, USA) linked to a GasBench II (Thermo Fisher Scientific, Poway, CA, USA). The δ^13^C value is relative to VPDB (Vienna Pee Dee Belemnite).

### Metagenomic sequencing and binning.

A total of 9 DNA samples, i.e., three layers (2, 15, and 30 cm below seafloor) of each push core, were extracted using DNeasy PowerSoil Pro kit (Qiagen, Germantown, MD, USA) according to the manufacturer’s protocol. DNA quality was measured using a Qubit double-stranded DNA (dsDNA) assay kit in a Qubit 2.0 fluorometer (Thermo Fisher Scientific, Waltham, MA, USA) and checked by 1% agarose gel electrophoresis. Sequencing libraries were generated using an NEBNext Ultra DNA library prep kit for Illumina (NEB, Ipswich, MA, USA) and sequenced using a NovaSeq 6000 platform (Illumina, San Diego, CA, USA). Clean data (150-bp paired-end reads) were obtained by removing adapters, barcodes, reads containing poly(N), and low-quality reads from the raw data.

Clean reads of the three samples from the same core were coassembled using MEGAHIT v.1.2.9 (83) with parameters “–k-min 27 –k-max 147 –k-step 12” and remapped to assemblies using Bowtie2 v.2.4.4 (84) with default settings to receive the coverage of contigs. Genomic binning was implemented using three programs, including MetaBAT2 v.2.12.1 (85), MaxBin2 v.2.2.7 (86), and CONCOCT v.1.1.0 (87), with 1.5 kb as the contig length cutoff. Furthermore, MAGs were refined using the “bin_refinement” module of MetaWRAP v.1.3 (88) and anvi’o v.7.1 (89). The quality and taxonomic information of MAGs were obtained using CheckM v.1.1.2 (90) and GTDB-TK v.1.6.0 (91), respectively.

### MAG dereplication and annotation.

All ANME archaeal MAGs, including 50 MAGs collected from public databases and 13 MAGs recovered in this study, were first dereplicated using the “dereplicate” module of dRep v.3.2.2 (92) with parameters “-comp 50 -con 10 –P_ani 0.9 –S_ani 0.99.” After dereplication, 21 ANME-1a/b, 11 ANME-2a/b, and 15 ANME-2c MAGs were retained for further analyses. The coding sequence of genomes was predicted using Prodigal v.2.6.3 with the “-p meta” parameter (93) and annotated against the Kyoto Encyclopedia of Genes and Genomes (KEGG) (94) and TransportDB v.2.0 databases (95) using Diamond v.2.0.4 (96) with coverage of >75% and E values of <1 × 10^−20^. KEGG pathways were reconstructed using the online KEGG Mapper (97). Functional genes involved in anaerobic methane oxidization and sulfate reduction were retrieved based on previous studies (29, 55, 78, 79, 98, 99). The statistical likelihoods for the presence or absence of sulfate reduction pathways in MAGs were assessed using MetaPOAP (100) with four marker genes, including *cysD*, *cysC*, *cysH*, and *fsr*. To identify potential *c*-type cytochrome containing the CXXCH motif, protein domains were predicted using DRAM v.1.2.4 (101) with the Pfam database (102), and the number of potential heme-binding sites was derived from the abundance of the CXXCH motif. Finally, the subcellular localization of multiheme *c*-type cytochromes was predicted using PSORTb v.3.0 (103).

### Phylogenetic and statistical analyses.

A maximum likelihood tree based on the multiple-sequence alignment of 122 archaeal marker proteins (104) was inferred using the Genome Taxonomy Database Toolkit (GTDB-Tk) v.1.5.0 (91). In brief, amino acid sequences of the genomes were predicted using Prodigal v.2.6.3 (93) and then aligned to Pfam and TIGRfam hidden Markov models using HMMER v.3.3 (http://hmmer.org/). The optimal model (JTT+F+G4) was then selected using ProtTest v.3.4.2 (105), and a phylogenetic tree was constructed using IQ-TREE v1.6.12 (106) with the ultrafast bootstrap parameter “-bb 1000” (107). Finally, the phylogenetic tree was visualized using iTOL v.5 (108).

The Shapiro-Wilk test was implemented using the “shapiro.test” function in R software (109) to test whether the abundance of iron transporters per genome, average number of hemes per *c*-type cytochrome, and GC content of MAGs were normally distributed. We applied nonparametric tests using “wilcox.test” to evaluate the differences among groups with abnormal distribution and “t.test” to evaluate the differences among groups with a normal distribution.

### Data availability.

Metagenomic sequences have been deposited in National Center for Biotechnology Information (NCBI) under BioProject no. PRJNA837106. All MAGs used in this study have been deposited in National Omics Data Encyclopedia (NODE) under project no. OEP002857. All bioinformatics commands are available via https://github.com/jchenek/scripts-for-methane-seep-ANME. We declare that all data supporting the findings of this study are available within the article and its supplemental material files or from the corresponding authors upon request.
