# Supplementary figures and images for: Genomic Insights into Niche Partitioning across Sediment Depth among Anaerobic Methane-Oxidizing Archaea in Global Methane Seeps
Source: mSystems. 2023 Mar 16;8(2):e01179-22. doi: 10.1128/msystems.01179-22 (PMC10134854; doi:10.1128/msystems.01179-22)

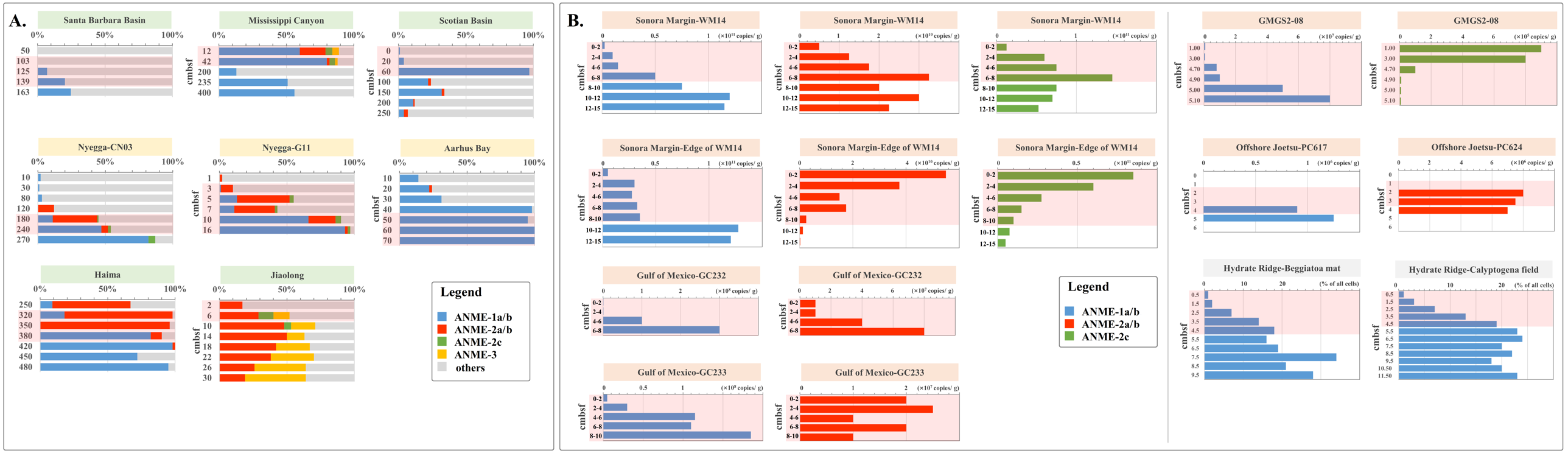

Supplement: FIG S1 [file msystems.01179-22-s0001.tif]
