# Supplementary material for: Genomic Insights into Niche Partitioning across Sediment Depth among Anaerobic Methane-Oxidizing Archaea in Global Methane Seeps
Source: mSystems. 2023 Mar 16;8(2):e01179-22. doi: 10.1128/msystems.01179-22 (PMC10134854; doi:10.1128/msystems.01179-22)
Supplement: TABLE S1 [file msystems.01179-22-s0002.docx]

**Supplementary Table S1.** Information of the methane seeps with ANME distribution pattern reported over the last two decades. ANME, anaerobic methanotrophic archaea.

| **Methane-seep Site** | **Longitude**  **(Degrees east)** | **Latitude**  **(Degrees north)** | **Depth (m)** | **Data type** | **Year** | **References** |
| --- | --- | --- | --- | --- | --- | --- |
| Eel River Basin | -124.60 | 40.79 | 526 | CARD-FISH | 2004 | (Orphan et al., 2004) |
| Gulf of Mexico | -91.28 | 27.72 | 650 | CARD -FISH | 2005 | (Orcutt et al., 2005) |
| Hydrate Ridge | -125.15 | 44.57 | 777 | CARD -FISH | 2005 | (Knittel Katrin et al., 2005) |
| Santa Barbara Basin | -119.99 | 34.23 | 587 | Amplicon | 2009 | (Harrison et al., 2009) |
| Offshore Joetsud | 138.05 | 37.52 | 1000 | qPCR | 2011 | (Yanagawa et al., 2011) |
| Nyegga G11 | 5.29 | 64.66 | 746 | Amplicon | 2011 | (Roalkvam et al., 2011) |
| Nyegga CN03 | 5.29 | 64.66 | 746 | Amplicon | 2012 | (Roalkvam et al., 2012) |
| Sonora Margin | -111.48 | 27.59 | 1574 | qPCR | 2013 | (Vigneron et al., 2013) |
| Mississippi Canyon | -88.49 | 28.86 | 879 | Amplicon | 2016 | (Underwood et al., 2016) |
| Haima | 110.41 | 16.73 | 1370 | Amplicon | 2017 | (Niu et al., 2017) |
| Aarhus Bay | 10.457 | 56.103 | 100 | Amplicon | 2019 | (Beulig et al., 2019) |
| GMGS2-08 | 118.00 | 22.00 | 798 | qPCR | 2019 | (Cui et al., 2019) |
| Jiaolong F3 site | 119.28 | 22.12 | 1162 | Amplicon | 2020 | (H. Li et al., 2020) |
| Scotian Basin | -60.21 | 43.01 | 2306 | Amplicon | 2020 | (Dong et al., 2020) |
