# Supplementary material for: Genomic Insights into Niche Partitioning across Sediment Depth among Anaerobic Methane-Oxidizing Archaea in Global Methane Seeps
Source: mSystems. 2023 Mar 16;8(2):e01179-22. doi: 10.1128/msystems.01179-22 (PMC10134854; doi:10.1128/msystems.01179-22)
Supplement: TABLE S8 [file msystems.01179-22-s0009.docx]

**Supplementary Table S8. A.** Shapiro-Wilk test results show if each group's numeric data were normally distributed. **B.** Evaluation of group differences. A nonparametric test (wilcox.test) was implemented to evaluate the differences among groups with abnormal distribution, and a t-test (t.test) was implemented to evaluate the differences among groups with normal distribution. NA, not applicable. ANME, anaerobic methanotrophic archaea.

| **A. Shapiro-Wilk test** | **1a/b** | **2a/b** | **2c** |
| --- | --- | --- | --- |
| Average number of haems per c-type cytochrome | 0.01724 | 0.9023 | 0.6877 |
| Number of flagella encoding genes per genome | 0.000194 | 0.03668 | 0.04338 |
| Number of *frh* genes per genome | 0.1208 | 0.02525 | NA |
| Number of *mvh* genes per genome | 0.01922 | 0.002564 | 0.003785 |
| GC content | 0.001062 | 0.001671 | 0.004548 |
| **B. Differences among groups** | **1a/b vs 2a/b** | **1a/b vs 2c** | **2a/b vs 2c** |
| Average number of haems per c-type cytochrome (t.test) | 0.0001684 | 0.0000001739 | 0.4625 |
| Number of flagella encoding genes per genome (wilcox.test) | 0.02912 | 0.05951 | 0.2075 |
| Number of *frh* genes per genome (t.test) | 0.0003537 | NA | NA |
| Number of *mvh* genes per genome (wilcox.test) | 0.558 | 0.01053 | 0.1475 |
| GC content (wilcox.test) | 0.5037 | 0.000001027 | 0.000009584 |
