# Supplementary material for: Genomic Insights into Niche Partitioning across Sediment Depth among Anaerobic Methane-Oxidizing Archaea in Global Methane Seeps
Source: mSystems. 2023 Mar 16;8(2):e01179-22. doi: 10.1128/msystems.01179-22 (PMC10134854; doi:10.1128/msystems.01179-22)
Supplement: TABLE S6 [file msystems.01179-22-s0007.docx]

**Supplementary Table S6.** Concentrations of nitrate and nitrite in Haima methane-seep sediments. cmbsf, cm below the seafloor.

| **cmbsf** | **Nitrate (mg/ L)** | **Nitrite (mg/ L)** |
| --- | --- | --- |
| 0 | 0.1120 | - |
| 2 | 0.0256 | - |
| 4 | - | - |
| 6 | - | - |
| 8 | - | - |
| 10 | - | - |
| 20 | - | - |
| 25 | - | - |
| 30 | - | - |
| 35 | - | - |
| 40 | - | - |

-: Lower than measurement limit.
